# Supplementary material for: Optimizing nitrogen removal with an immobilized biological filler system: realizing stage-independent operational process
Source: PLoS One. 2025 Mar 5;20(3):e0315864. doi: 10.1371/journal.pone.0315864 (PMC11882092; doi:10.1371/journal.pone.0315864)
Supplement: S1 File — (DOCX) [file pone.0315864.s001.docx]

Research on the performance of immobilized biological filler nitrogen removal system -- Realizing independent operation at each stage

Xuyan Liu^1,2¶^, Hong Yang^3^, Jiawei Wang^4*^

**^1^**Hebei GEO University, Shijiazhuang, China;

^2^Hebei Center for Ecological and Environmental Geology Research, Hebei GEO University, Shijiazhuang, China;

^3^Key Laboratory of Beijing Water Quality Science and Water Environment Recovery Engineering, Beijing University of Technology, No. 100, Pingleyuan, Chaoyang District, Beijing, China;

^4^ Hebei Key Laboratory of Water Quality Engineering and Comprehensive Utilization of Water Resources, Hebei University of Architecture, Zhangjiakou, China;

*Corresponding author

E-mail: [ykaisjz@163.com](mailto:ykaisjz@163.com) (WJW)


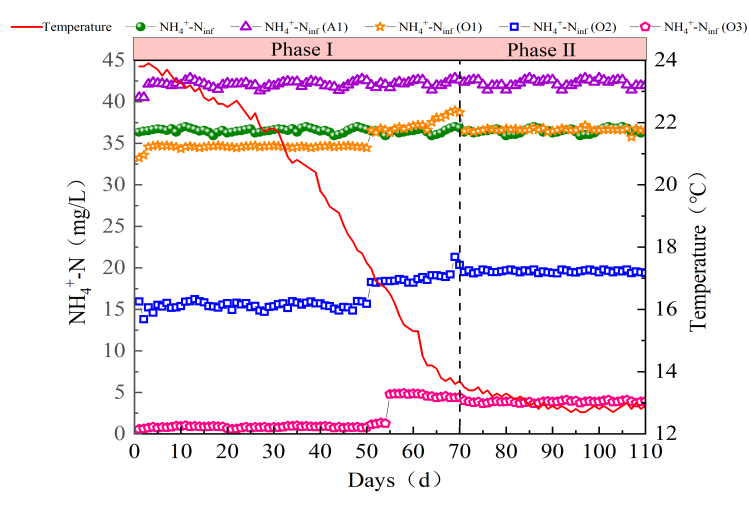

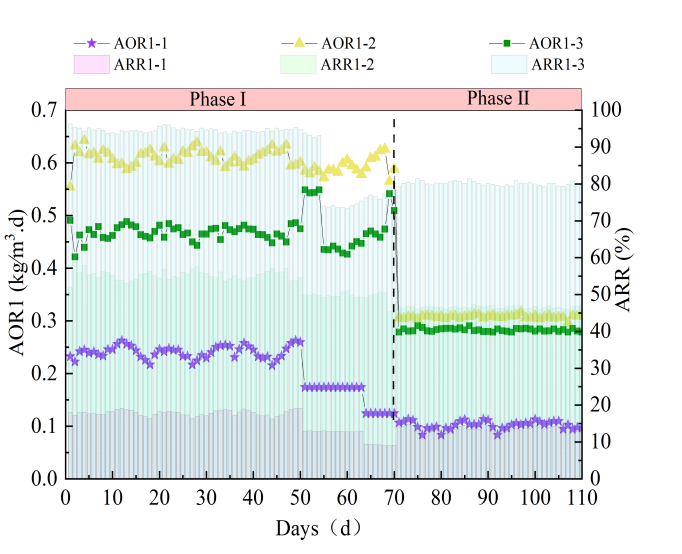

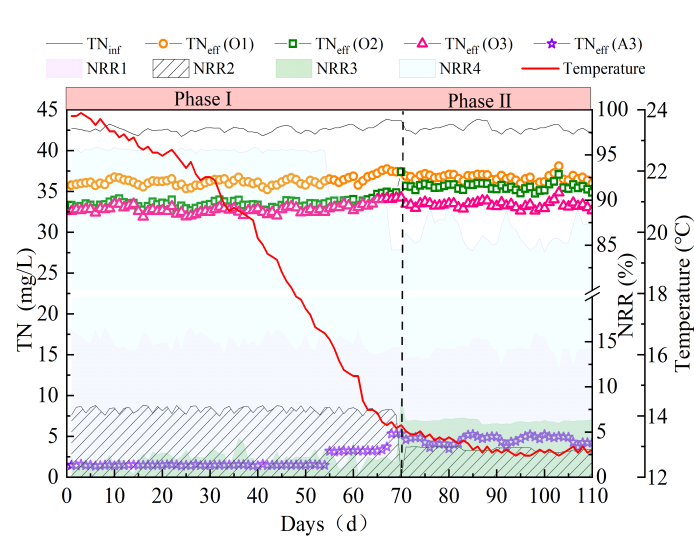

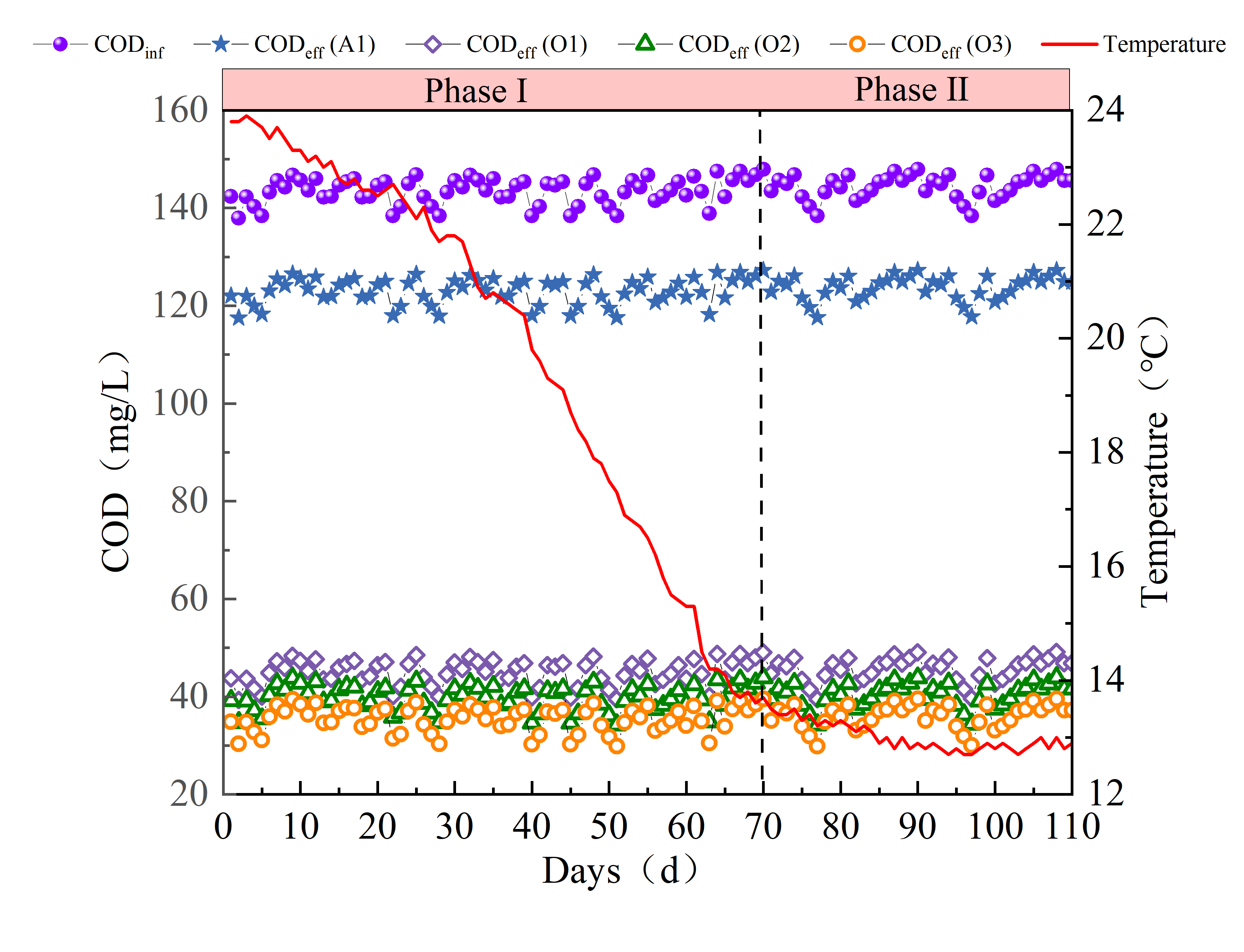


**S1 Fig. Stage performance of nitrification stage under the influence of organic matter: (a) Changes in NH_~~4~~_^+^-N concentration; (b) AOR and ARR; (c) TN change and removal rate; (d) COD concentration change**

By comparing the nitrification performance of AOA and AAOA system, the influence of organic matter entering nitrification unit on nitrification performance of filler was explored.

The seasonal temperature variation and low temperature operation stage data were selected for research. It can be seen from S1 Fig(a)(b) that AOR and ARR of each nitrification unit remained stable with the seasonal decrease of temperature (day 1-70).According to equation 1, at this time, AOR ranges from 0.23 to 0.24, 0.59 to 0.60, 0.47 to 0.48 kg/m^3^.d, ARR ranges from 17.9 to 18.2, 54.17 to 53.92, and 93.2 to 94.5%, respectively, and NH_4_^+^-N in the effluent of O3 can be stabilized below 1 mg/L. However, with the continuous decrease of temperature, the performance of nitrification stages began to decline when the temperature reached below 17℃, and the AOR1-1 decreased to 0.16~0.17 kg/m^3^.d, the AOR1-2 remained at 0.58~0.59 kg/m^3^.d, and the AOR1-3 decreased to 0.43~0.44 kg/m^3^.d. ARR decreases from 12.94 to 13.04, 49.97 to 50.14 and 73.2 to 74.08%, respectively, while NH_4_^+^-N in the effluent of O3 gradually increases to 4.42 mg/L (day 70).When the adjustment working condition enters the low temperature stage, AOR1-1 decreases to 0.124-0.126 kg/m^3^.d, AOR1-2 to 0.302~0.0.307 kg/m^3^.d, and AOR1-3 to 0.27~0.28 kg/m^3^.d. ARR1-2 decreased to 45.91-46.5% while ARR1-3 increased to 79.7~80.9%.

Therefore, it can be shown that the decrease of temperature has a significant impact on the performance of O1. Once the temperature decreases, AOR1-1 will decrease significantly, while the changes of AOR1-2 and AOR1-3 are not obvious. Compared with the parameters below 17℃, it is found that ARR1-2 and ARR1-3 will increase to a certain extent when the performance of O1 decreases, indicating that they can still maintain relatively good performance. According to the change of COD, it can also be seen that due to the role of heterotrophic bacteria in raw water, most of the organic matter in the influent is consumed by heterotrophic bacteria in O1 and competes with nitrifying bacteria DO. At this time, the effluent COD decreases from 143.2~145.3 mg/L in raw water to 46.3~ 43.5mg /L. This is also why O1 performance cannot be improved and it is difficult to resist low temperatures. On the other hand, COD in O2 and O3 has been reduced to a low level, so the performance is not affected, and due to the poor performance of O1, the concentration entering the last two units increases, thus increasing the subsequent ammonia oxidation rate.
