# Supplementary material for: Optimizing nitrogen removal with an immobilized biological filler system: realizing stage-independent operational process
Source: PLoS One. 2025 Mar 5;20(3):e0315864. doi: 10.1371/journal.pone.0315864 (PMC11882092; doi:10.1371/journal.pone.0315864)
Supplement: Attachment — Submitted filename: Response to Reviewers.docx [file pone.0315864.s002.docx]

**Response to** **Reviewer**

Dear Editors and Reviewers:

Thank you for your letter and for the reviewers’ comments concerning our manuscript entitled **The performance of an immobilized biological filler nitrogen removal system -- Realizing independent operation at each stage (manuscript number: PONE-D-24-36163)**. All comments are valuable and very helpful for revising and improving our paper. We have carefully studied the comments and have revised our manuscript accordingly. The main corrections in the manuscript and the responds to the reviewer’s comments are as following:

**To Reviewer #1**

Thank you very much for pointing out these issues. We have revised our manuscript accordingly.

**Comments:**

1. ***Highlight points should be presented.***

Thank you for this comment. We add Highlight to the submission as follows: “1. An independent biological nitrogen removal system based on immobilized fillers was proposed; 2. The efficient separation of functional bacteria for denitrification of municipal sewage was realized; 3. The process system can meet the requirements of "Surface water Environmental Quality Standard" (GB-3838-2002) quasi Class IV; 4. An independent hydrolyzing and acidizing device composed of immobilized fillers was constructed with high performance. ”

***2. Line 26: AAOA nitrogen removal system. Please use the full term in its first appear. Use “Anaerobic - anoxic - aerobic - anoxic (AAOA)” instead of using abbreviations.***

Thank you for this comment; we have revised it.

The revised section now reads as follows:

“**Abstract**

Immobilized fillers, including hydrolytic-acidification, nitrification and denitrification, were prepared in this study to improve the nitrogen removal performance of the traditional activated sludge process. An anaerobic-anoxic-aerobic anoxic nitrogen removal system was constructed to effectively separate the functional bacteria that remove nitrogen.” Revised in the manuscript **Abstract**, shown in the underlined section.

***3. Proofreading is essential as there are many mistakes. Please get the language of the study checked by a native English language speaker.***

Thank you for this comment. It is our negligence and we are sorry about this. We had a native English speaker proofread the manuscript's language.

***4. The knowledge gap has not been indicated properly in the introduction.***

Thank you for this comment. We have made a major revision to the abstract, please refer to it.

The revised section now reads as follows:

**Abstract**

Immobilized fillers, including hydrolytic-acidification, nitrification and denitrification, were prepared in this study to improve the nitrogen removal performance of the traditional activated sludge process. An anaerobic-anoxic-aerobic anoxic nitrogen removal system was constructed to effectively separate the functional bacteria that remove nitrogen. The correlation and influence between each unit were explored by studying the operating performance of the system. The levels of ammonia nitrogen and total nitrogen in the effluent could be stabilized at 0.75–0.83 and 1.5–2 mg/L, respectively, when the total hydraulic retention time (HRT) of the system was 6.4 h and the nitrification unit HRT was 3 h. These parameters improved significantly compared with the traditional process of activated sludge. The unit performance study showed that a reduction in the time of hydrolytic-acidification to 0 min resulted in an increase in the levels of nitrite nitrogen and nitrate nitrogen in the effluent of unit A2 to 6.11 ± 0.2 mg/L and 3.67 ± 0.1 mg/L, respectively. Thus, a highly active hydrolysis-acidification stage is the prerequisite for A2 to fully utilize the raw organic matter in the water to remove nitrogen. When the raw organic matter in the water entered the O1 unit directly (without an A2 unit), the ammonia oxidation rate (AOR) decreased significantly (from 0.32–0.33 to 0.22–0.23 kg/m^3^⋅d). At a low temperature, the AOR of O1 unit decreased even more (to 0.11–0.12 kg/m^3^⋅d). At this time, the AOR that was not influenced by organic matter only decreased slightly. This indicates that causing the organic matter to enter the nitrification stage is essential to maintain its stability and resist low temperatures. Revised in the manuscript **Abstract**, shown in the underlined section.

***5.Novelty of the studied is not well-established***

Thank you for this comment. The anaerobic- Anoxic-aerobic anoxic (AAOA) nitrogen removal system established in this manuscript is innovative for the following reasons:

Traditional activated sludge process is one of the most widely used processes in urban sewage treatment plants due to its wide application range and mature technology. However, due to the operation mode of the single sludge system, various functional bacteria (hydrolytic acidification, nitrification, denitrification removal bacteria) with different physiological characteristics and generation cycle exist in the same system for reaction, which makes it difficult to give full play to their respective advantages, and makes it difficult to improve the overall performance of the process (Zanetti et al., 2017; Wang et al., 2014). This results in many problems, such as low biochemical efficiency, poor stability, low utilization rate of organic matter in raw water and unable to give full play to biological nitrogen and phosphorus removal efficiency. Therefore, the development of nitrogen removal systems independently carried out by various functional bacteria has become a new trend of municipal sewage treatment.

In this study, the hydrolytic-acidification unit with stable anaerobic state was established by using hydrolytic-acidification immobilized filler (Wang et al., 2021). The method not only stores a high concentration of active biomass, but also provides a stable environment for the growth of hydrolyzed acidifying bacteria. On the basis of improving the insufficient activated sludge process, hydrolytic-acidification can be fully utilized without affecting the design of the subsequent process, aiming to provide a more stable and effective new method for the full utilization of organic matter in the raw water of municipal sewage and TN removal. Then, the AAOA nitrogen removal system was established by hydrolytic-acidification, nitrification and denitrification immobilized fillers to achieve effective separation of various functional bacteria. The performance of the system was analyzed, studied and improved through process regulation, providing an effective method for efficient biological nitrogen removal of municipal sewage.

**Reference**

[1] Zanetti L, Frison N, Nota E, et al. Progress in real-time control applied to biological nitrogen removal from wastewater. A short-review[J]. Desalination, 2012, 286(2012): 1-7.
[2] Wang Y, Li W G, Irini A, et al. Removal of organic pollutants in tannery wastewater from wet-blue fur processing by integrated Anoxic / Oxic (A/O) and Fenton: Process optimization[J]. Chemical Engineering Journal, 2014, 252: 22-29.

[3] Wang J, Fan Y C, Chen Y P. Nitrogen removal performance and characteristics of gel beads immobilized anammox bacteria under different PVA: SA ratios[J]. Water environment research, 2021, 93(9): 1627-1639.

***6.Include a nomenclature section before introduction.***

Thank you for this comment. We added a nomenclature section before the introduction.

The revised section now reads as follows:

The list of self-named abbreviations

| AAOA | Immobilized filler nitrogen removal system (with A2) |
| --- | --- |
| AOA | Immobilized filler nitrogen removal system (no A2) |
| A1 | Hydrolytic-acidification unit |
| A2 | Primary denitrification unit |
| O | Nitrification unit |
| A3 | Secondary denitrification unit |

Common Abbreviations

| TN | Total Nitrogen |
| --- | --- |
| NH_4_^+^ -N | Ammonia Nitrogen |
| NO_2_^-^-N | Nitrite Nitrogen |
| NO_3_^-^-N | Nitrate Nitrogen |
| COD | Chemical Oxygen Demand |
| DON | Dissolved Organic Nitrogen |
| DO | Dissolved Oxygen |
| HRT | Hydraulic Retention Time |
| AOR | Ammonia Oxidation Rate |
| ARR | Ammonia Nitrogen Removal Rate |
| NRR | TN Removal Rate |
| SEM | Scanning electron microscopy |
| VFAs | Volatile Fatty Acids |

***7. You cited "old references" not too many of them were printed after 2017.***

Thank you for this comment. We have updated the references in the manuscript .

8. ***In order to establish the significance and value of the study, it is necessary to provide a comprehensive rationale for the research, which emphasizes its relevance and unique contributions to the current scholarly discourse. This will strengthen the study's originality and scholarly impact. The following publications related to biological N removal are useful suggested studies to get the benefit to update this part***

***I. Integrated electro-anammox process for nitrogen removal from wastewater***

***II. Simultaneous nitrification and denitrification by controlling current density and dissolved oxygen supply in a novel electrically-induced membrane bioreactor***

***III. Modification of nitrifying microbial community via DC electrical field application***

***IV. Coupling membrane electro-bioreactor with anammox process to treat wastewater at low temperatures***

Thank you for this comment. We think the references you provided are very valuable and we have cited them in the manuscript according to the content.

9. ***All figures should be optimized.***

Thank you for this comment. We have optimized pixel and font sizes for all figures.

10. ***The figure captions should be written with more informative.***

Thank you for this comment. We have optimized the figure captions.

The revised section now reads as follows:

Fig 1. Schematic diagram of the Anaerobic - anoxic - aerobic - anoxic (AAOA) nitrogen removal process.

Fig. 2. Schematic diagram of the process changes under the influence of organic matter, including the AOA and AAOA nitrogen removal processes.

Fig 3. AAOA system optimized reflux ratio stage performance: (a) Changes in NH_4_^+^-N; (b) AOR and ARR; (c) Changes in TN ( (AAOA: Anaerobic - anoxic - aerobic - anoxic; NH_4_^+^-N: Ammonia nitrogen; AOR: Ammonia oxidation rate; ARR: Ammonia removal rate).

Fig. 4. Optimization of the HRT stage performance in the AAOA system: (a) Changes in NH_4_^+^-N concentration; (b) AOR and ARR;(c) Changes in TN (AAOA: Anaerobic - anoxic - aerobic - anoxic; HRT: Hydraulic Retention Time; NH_4_^+^-N: Ammonia nitrogen; AOR: Ammonia oxidation rate; ARR: Ammonia removal rate).

Fig 5. Performance of the AAOA system at different stages of temperature changes: (a) Changes in the NH_4_^+^-N concentration; (b) Ammonia oxidation rate and removal rate; (c) Changes in the TN concentration (AAOA: Anaerobic - anoxic - aerobic - anoxic; NH_4_^+^-N: Ammonia nitrogen; TN: Total nitrogen).

Fig 6. Nitrogen variation and denitrification contribution of each unit of the AAOA system (AAOA:Anaerobic - anoxic - aerobic - anoxic).

Fig 7. A comparison of the impact of organic matter on the nitrification performance between the AAOA and AOA systems.

Fig 8. Effect of hydrolytic acidification on the performance of a primary denitrification unit.

Fig 9. SEM images of immobilized fillers: (a) nitrification filler magnified by 2k times; (b) nitrification filler magnified by 5 k times; (c) nitrification filler magnified by 10 k times; (d) nitrification filler magnified by 20 k times; (e) denitrification filler magnified by 2 k times; (f) denitrification filler magnified by 5 k times; (g) denitrification filler magnified by 10 k times; (h) denitrification filler magnified by 20 k times (SEM: Scanning Electron Microscopy).

Fig 10. The bacterial community structure of the denitrification unit before and after the AAOA system.

Fig 11. The bacterial community structure of the nitrification unit in the AAOA system.

11. ***The conclusion should be rewritten. For example, this section “We explored the performance of an AAOA nitrogen removal system constructed by hydrolytic acidification, nitrification, and denitrification immobilized fillers, with the following conclusions: When the total HRT of the system is 6.4 h and the nitrification unit HRT is 3 h, the effluent NH4+-N can be stabilized at 0.78–0.84 mg/L, 551 and the TN can reach 1.5–2 mg/L.” I don't think this section is clear and grammatically accurate.***

Thank you for this comment. We have rewritten the conclusion.

The revised section now reads as follows:

**Conclusion**

This study examined the performance of an AAOA nitrogen removal system constructed by hydrolytic acidification, nitrification and denitrification immobilized fillers. The overall operation of the system under the background of municipal sewage was studied, including the effects of reflux ratio, HRT and seasonal temperature on the operation of the system. The following conclusions were drawn:

1. Compared with the other existing biological nitrogen removal processes, AAOA removed nitrogen more effectively. When the total HRT of the system is 6.4 h and the nitrification unit HRT is 3 h, the effluent NH_4_^+^-N can be stabilized at 0.78–0.84 mg/L. The TN can reach 1.5–2 mg/L. When the system R is 90%, the AOR of O1 can be increased by 0.33–0.35 kg/m^3^⋅d; the effluent COD of A1 is 45.5-48.3 mg/L, and the NH_4_^+^-N can be kept below 1 mg/L. At low temperatures in the winter (<14 ℃), the NH_4_^+^-N and TN of the effluent of AAOA system can still reach the national standard Class A standard.
2. Organic matter can still affect the performance of the nitrification unit. Although the nitrification filler contains highly efficient nitrifying bacteria, the heterotrophic bacteria in sewage will affect the oxidation of NH_4_^+^-N when there is abundant organic matter. Therefore, first-stage denitrification is necessary in the AAOA system, which is particularly reflected during the process of quarterly temperature changes. Above 17 ℃, the AOR and ARR of AOA were 0.22–0.23 kg/m^3^⋅d and 17.5%, respectively, while the AOR and ARR of the AAOA were 0.32–0.33 kg/m^3^⋅d and 8.7%, respectively. When the temperature decreased to 17 ℃ or below, the AOR and ARR of AOA decreased to 0.11–0.12 kg/m^3^⋅d and 13.0%, respectively, while the AAOA only slightly decreased.
3. The change in the organic composition of raw water through the experimental results of HRT change of A1 is crucial to improving the performance of denitrification. Changing the HRT of the A1 (0, 30, 60 min) to an HRT of 60 min results in effective, A2, and the effluent NO_2_^-^-N and NO_3_^-^-N concentrations are maintained at a low level.

**Special thanks to you for all your valuable comments!**

**To Reviewer #2**

Thank you very much for your comments and suggestions. We have modified and explained your question.

**Comments:**

1. ***Abstract:It is noted that the abstract is very brief and should be enriched to show the problem solved and the novelty of the research. Moreover, the abstract does not show the methodology used throughout the article.***

Thank you for this comment. We have rewritten the Abstract and highlighted it with underline and yellow.

The revised section now reads as follows:

**Abstract**

Immobilized fillers, including hydrolytic-acidification, nitrification and denitrification, were prepared in this study to improve the nitrogen removal performance of the traditional activated sludge process. An anaerobic-anoxic-aerobic anoxic nitrogen removal system was constructed to effectively separate the functional bacteria that remove nitrogen. The correlation and influence between each unit were explored by studying the operating performance of the system. The levels of ammonia nitrogen and total nitrogen in the effluent could be stabilized at 0.75–0.83 and 1.5–2 mg/L, respectively, when the total hydraulic retention time (HRT) of the system was 6.4 h and the nitrification unit HRT was 3 h. These parameters improved significantly compared with the traditional process of activated sludge. The unit performance study showed that a reduction in the time of hydrolytic-acidification to 0 min resulted in an increase in the levels of nitrite nitrogen and nitrate nitrogen in the effluent of unit A2 to 6.11 ± 0.2 mg/L and 3.67 ± 0.1 mg/L, respectively. Thus, a highly active hydrolysis-acidification stage is the prerequisite for A2 to fully utilize the raw organic matter in the water to remove nitrogen. When the raw organic matter in the water entered the O1 unit directly (without an A2 unit), the ammonia oxidation rate (AOR) decreased significantly (from 0.32–0.33 to 0.22–0.23 kg/m^3^⋅d). At a low temperature, the AOR of O1 unit decreased even more (to 0.11–0.12 kg/m^3^⋅d). At this time, the AOR that was not influenced by organic matter only decreased slightly. This indicates that causing the organic matter to enter the nitrification stage is essential to maintain its stability and resist low temperatures.

***2. English and editing:***

***The paper needs careful editing by someone with expertise in technical English editing who pays particular attention to English grammar, spelling, and sentence structure so that the study's goals, methods, and results are clear to the reader.***

Thank you for this comment. We have found professional language and culture local people to polish our manuscripts.

***3. Figures:***

***The figures are too small to read and recognize the results, so please maximize and refine the shown figures. Please show the comparison and differences between the conventional methods and the paper method for removal of nitrogen. (The paper is poor for showing the literature review and previous work).***

Thank you for this comment. We have modified the figures according to your request.

**The comparison and differences between the conventional methods and the paper method for removal of nitrogen：**

**The conventional methods：**

The traditional activated sludge process is widely used because of its mature technology, simple operation and stable treatment effect. The operation core of this process is mainly four types of common functional bacteria in sewage treatment - hydrolytic acidifying bacteria, nitrifying bacteria, denitrifying bacteria and phosphorous accumulating bacteria. Through the operation mode of a single mud system, the pollutants are removed through three treatment units of anaerobic, anoxic and aerobic, and finally enter the sedimentation tank for solid-liquid separation and collection of bacteria, and then return to the front end of the reaction for biomass maintenance. This mode of operation directly leads to bacteria with different physiological characteristics and generation cycles working in the same system, so it is difficult to play their respective advantages, making the overall process biochemical efficiency is low. This is related to the poor utilization rate of organic matter in raw water, the low efficiency of nitrification and the inability to give full play to the biological phosphorus removal efficiency. Meanwhile, the process also has some limitations, such as high energy consumption, large sludge production, and limited effectiveness in the removal of certain pollutants. Therefore, with the progress of technology, many improved activated sludge processes such as sequencing batch activated sludge process (SBR) and membrane bioreactor (MBR) have emerged successively to improve the efficiency and quality of sewage treatment.

**The paper method for removal of nitrogen and its advantages over conventional methods：**

The immobilized microorganism process is also more and more applied to the field of wastewater treatment because of its unique advantages. In sewage treatment, immobilization technology usually uses a variety of measures to screen and culture the corresponding microorganisms, and then fixes them by different methods after enriching the dominant strains. The fixed microbial body usually has many advantages such as high density of bacteria, high treatment efficiency, fast reaction speed, strong stability, easy solid-liquid separation and less residual sludge production, and can reduce secondary pollution.

In order to improve the traditional nitrogen removal process, this study used the mature immobilization technology of our research group to prepare hydrolytic acidification, nitrification and denitrification immobilization fillers, and constructed an AAOA nitrogen removal system composed of hydrolytic acidification unit (A1), primary denitrification unit (A2), nitrification unit (O1-O3) and secondary denitrification unit (A3).

AAOA nitrogen removal process is built on the basis of a large number of studies on the application of immobilized fillers to municipal wastewater, and based on the key problems that need to be solved by traditional process. Firstly, to solve the problem of low nitrification efficiency of traditional process, efficient nitrification immobilized filler is used to improve its efficiency. In the study, it was found that organic matter and heterotrophic bacteria brought by raw water would also affect the performance of the nitrification stage of the process. Therefore, efficient denitrification immobilized filler was placed at the front end of the nitrification stage to make full use of organic matter in raw water and avoid heterotrophic bacteria competing with nitrifying bacteria for dissolved oxygen in the presence of organic matter and affecting its performance. Secondly, the study found that the anaerobic hydrolytic acidification of raw water organic matter is the key premise of denitrification to make full use of raw water organic matter. Therefore, hydrolytic acidification immobilized filler was used to improve the hydrolytic acidification performance in anaerobic stage to enhance the utilization of raw water organic matter by denitrification.

***4. Could you please explain how and why you chose the values of the different important parameters?***

Thank you for this comment.

The reason why we choose these parameters is that during the operation of the nitrogen removal system, there are mainly three factors that will affect its performance, namely HRT (hydraulic retention time), reflux ratio and seasonal temperature change.

HRT is an important parameter in water treatment process. It represents the average residence time of the sewage in the reactor, during which the pollutants in the sewage interact with the microorganisms in the bioreactor to carry out biochemical reactions. HRT is calculated as follows: HRT = V/Q, where V represents the effective volume of the reactor (cubic meters), and Q represents the inlet flow rate (cubic meters/hour). The setting of HRT has an important influence on the treatment effect of wastewater. Too short HRT may lead to incomplete biochemical reaction and weak treatment degree. Too long HRT may lead to aging of sludge and increase the economic burden. Therefore, when we study innovative nitrogen removal processes, we need to first consider the impact of HRT on system performance.

‌ Sewage return ratio refers to the ratio of the sewage return flow rate to the water intake of the aeration tank. This ratio is usually indicated by the letter R and is specifically the ratio of the return sludge flow in the aeration tank to the inflow flow ‌. In the process of sewage treatment, the size of reflux ratio has an important influence on the treatment effect. When the inflow water quality changes, it is hoped to adjust the reflux ratio to adapt to these changes, but because there is a certain time lag in the sewage treatment process, adjusting the reflux ratio is not immediately effective, usually takes a few hours to reflect the effect. Therefore, under normal circumstances, the reflux ratio remains constant, but in response to emergencies, adjusting the reflux ratio is an effective emergency means. Therefore, we need to discuss the size of reflux ratio in the AAOA nitrogen removal system to judge the performance and energy consumption of the system.

Seasonal temperature variation has significant influence on the performance of nitrogen removal system. ‌ Temperature fluctuations will lead to changes in microbial metabolic activities, which will affect the efficiency of the nitrogen removal system. For example, when the temperature is lowered from 35℃ to 25℃, the anaerobic ammonia oxidation (Anammox) nitrogen removal rate in the anaerobic zone will decrease, resulting in a significant decrease in the overall nitrogen removal rate ‌. In addition, temperature reduction will also affect the activity of nitrifying bacteria and denitrifying bacteria, and thus affect the nitrogen removal efficiency ‌. Specifically, the influence of temperature change on the nitrogen removal system is mainly reflected in the following aspects: The nitrification process is affected ‌ : Nitrifying bacteria and nitrifying bacteria are more sensitive to temperature, and the optimal growth temperature is 25-30℃. When the temperature is lower than 15℃, the nitrification rate will be sharply reduced, resulting in excessive ammonia nitrogen ‌. The denitrification process is affected ‌ : The suitable temperature of denitrifying bacteria is 20-35℃, when it is lower than 15℃, the reproductive rate and metabolic rate of denitrifying bacteria will decrease, resulting in the decrease of nitrogen removal effect ‌. Changes in microbial community structure ‌ : changes in temperature will cause changes in microbial community structure. For example, the abundance of anaerobic ammonia-oxidizing bacteria (AnAOB) will change with changes in temperature, which will affect the nitrogen removal efficiency‌. Therefore, we need to explore the adaptability of AAOA nitrogen removal system to seasonal temperature.

‌

***5. The equations should be written using and following the journal guidelines?***

Thank you for this comment.We have modified the equations according to the journal guidelines.

6. ***Please use subscript and superscript while editing “for example O3 not O3”***

Thank you for this comment. The unit of AAOA nitrogen removal system in this manuscript is named by itself to better explain the nature of each unit. The O3 you mentioned is the third reactor in the aerobic unit, so it is named O3 by itself, which is not suitable for subscript or superscript. We reviewed other subscripts or superscripts that must be executed*.*

The list of self-named abbreviations

| AAOA | Immobilized filler nitrogen removal system (with A2) |
| --- | --- |
| AOA | Immobilized filler nitrogen removal system (no A2) |
| A1 | Hydrolytic-acidification unit |
| A2 | Primary denitrification unit |
| O1 | Nitrification unit 1 |
| O2 | Nitrification unit 2 |
| O3 | Nitrification unit 3 |
| A3 | Secondary denitrification unit |

7. ***Line 98, “These not this”.***

Thank you for this comment. We've modified it, underlined it and highlighted it in yellow at Line 104.

8. ***Line 114, please revise.***

Thank you for this comment. You may feel that the preparation process of immobilized fillers is too general, I am very sorry to bring you a bad reading experience, because the preparation process is confidential, so there is no way to explain too much. We've modified it, underlined it and highlighted it in yellow at Line 122. The following figure is the physical picture of the immobilized filler.

9. ***Line 319, “decreased not was decreased”.***

Thank you for this comment. We've modified it, underlined it and highlighted it in yellow at Line 336.

10. ***Line 353, please revise.***

Thank you for this comment. We've modified it, underlined it and highlighted it in yellow at Line 370.

11. ***How did you get or measure the characteristics of the wastewater?***

Thank you for this comment. The AAOA immobilized filler nitrogen removal pilot system we created was set in Beijing Gaobeidian Sewage Treatment Plant, and the effluent from the initial sedimentation tank was used as the influent water of the system. Therefore, the performance of the nitrogen removal system we explored was based on actual sewage, which could better reflect the actual operating performance of the system. The characteristics of wastewater are measured in real time every day, which is the most accurate data every day.

12. ***What is PVA, I suggest you write abbreviations.***

Thank you for this comment. Polyvinyl alcohol (PVA) is a water-soluble polymer with a large number of hydroxyl groups on its surface, which can interact with biological macromolecules through hydrogen bonds and other chemical bonds, so as to realize the immobilization of biological macromolecules. The immobilization method of polyvinyl alcohol has good biocompatibility, stability and reusability, and is widely used in the preparation of bioimmobilization fillers. We've modified it, underlined it and highlighted it in yellow at Line 118.

13. ***Please revise section 2.1.***

Thank you for this comment. We've modified it.

The revised section now reads as follows:

The influent of the AAOA nitrogen removal pilot system originated from the primary sedimentation tank of the Beijing Gaobeidian Sewage Treatment Plant (Beijing, China). Table 1 provides an overview of the water quality of the influent.Revised in the manuscript **2.1 Experimental water quality**, shown in the underlined and yellow section at **Line 108-110**.

14. ***The innovation and advantages of the method or your work need to be emphasized and summarized whether in Introduction or Methodology.***

Thank you for this comment. We've modified it.

The revised section now reads as follows:

This study utilized urban sewage as the research object. It used hydrolytic acidification, nitrification and denitrification immobilized fillers to build an anaerobic-anoxic-aerobic anoxic (AAOA) pilot nitrogen removal system. This system enabled the different functional bacteria to separate and fully utilize their respective properties. It also solved the problems of the poor rate of utilization of the influent organic matter and low nitrogen removal efficiency of the traditional activated sludge process. The objectives were as follows: (1) to determine the overall operation performance of the AAOA nitrogen removal system under different working conditions and seasonal temperature changes, along with the key control parameters; (2) to determine the characteristics of each unit, including the influence of hydrolytic acidification on the first-order denitrification performance and organic matter on nitrification performance; and (3) to analyze the internal structure of each unit filler. These results provide a basis for the efficient and stable removal of nitrogen from municipal sewage. Revised in the manuscript **Introduction**, shown in the underlined and yellow section at **Line 92-105**.

15. ***I can tell that the authors did not follow the Journal guidelines for the structure of the sections and the references.***

Thank you for this comment. We've modified it.

16. ***The references are few compared with the importance of the topic. Please try to double the references at least with more recent references.***

Thank you for this comment. We've modified it.

**Special thanks to you for all your valuable comments!**

**To Reviewer #3**

1. ***But the data presented does not support all the results and does not make it possible to repeat the experiment. Especially the Materials and Methods section is incomplete. It does not state how the fillers where made in detail or how and what bacteria where immobilised in them. Similarly the reactors set up and operation is not stated in detail and any dimensions are missing.***

Thank you for this comment. You may feel that the preparation process of immobilized fillers is too general, I am very sorry to bring you a bad reading experience, because the preparation process is confidential, so there is no way to explain too much. The following figure is the physical picture of the immobilized filler. The construction parameters of the reactor have been modified.

The revised section now reads as follows:

Polyvinyl alcohol (PVA) was weighed and dissolved in water. It was then mixed with the different bacteria, and auxiliary additives were added to form a relatively viscous embedded solution. It should be noted that the different bacterial auxiliary agents were added in different ways. The equipment was developed in this laboratory to pressure the concentrated mixed liquid to form a hollow cylinder and cut it into small sections. These sections were then placed in a solution of saturated boric acid for crosslinking and stabilization. Finally, they were cleaned with water to prepare immobilized fillers of hydrolytic acidification, nitrification, and denitrification. The filler had no skeleton and was a hollow cylinder approximately 1 cm wide and 2 mm thick. Revised in the manuscript **Preparation of the immobilized filler**, shown in the underlined and yellow section at **Line 119-128**.

The AAOA immobilized filler nitrogen removal system consisted of hydrolytic acidification, nitrification, and denitrification fillers, They were the components of a hydrolytic acidification unit (A1; effective volume 200 L ), a primary denitrification unit (A2; effective volume 320 L), a nitrification unit (O1-O3; effective volume 200 L×3), and a secondary denitrification unit (A3; effective volume 160 L), The total volume was 1.28 m^3^. Revised in the manuscript **Reactor setup and operation**, shown in the underlined and yellow section at **Line 130-135**.

The data in this study are all the real data of AAOA nitrogen removal system operating under different working conditions and seasonal temperature changes based on actual municipal sewage, which is reproducible.

2. ***The discussion of the results could be much more in depth and next to the SEM images studying the microbial community in the fillers would give a much better understanding the system.***

Thank you for this comment. We have analyzed the community structure of each unit of the AAOA nitrogen removal system, but it has not been discussed due to too much content, and now we have revised it.

**Community structure analysis of the AAOA system**

**Comparative analysis of the community structure in primary and secondary denitrification**

Figure 10 primarily shows the abundance of bacteria that are involved in primary and secondary denitrification in the AAOA system and analyzes the differences in community structure during the long-term operation of denitrification fillers with different carbon sources. It was apparent that the denitrifying bacteria in A2 and A3 primarily included *Arcobacter* and *Thauera*, *Comamonas*, *Thiobacillus*, *Pseudomonas, Azoarcus, Flavobacterium, Defluviimonas,* and *Thermomonas* among others, There was little difference between the two categories. However, there was a difference in abundance. In A2 and A3, *Arcobacter, Thauera* and *Comamonas* predominated, and the abundance of *Arcobacter* was 14.34 and 21.23%, respectively. *Pseudomonas* and *Thauera* were found to be typical concurrent autotrophic denitrifying bacteria, while *Arcobacter, Thiobacillus and Azoarcus* were the primary autotrophic denitrifying microorganisms. We previously used high-throughput sequencing to show that *Thauera* and *Paracoccus* in the denitrifying immobilized filler were the predominant bacterial genera. The abundance of denitrifying bacteria commonly found in wastewater treatment plants, such as *Pseudomonas* and *Comamonas*, increased significantly with the operation in actual sewage. The denitrification properties of *Flavobacterium, Defluviimonas* and *Thermomonas* have also been verified, and these three bacteria are more abundant in A3 than A2.

In addition to the dominant denitrifying bacteria in A2 and A3, some bacteria with a hydrolytic-acidification function were also found. The primary species of bacteria were *Ornatilinea, Longilinea, Ignavibacterium,* and *Anaerolinea*. *Ornatilinea* has been shown to ferment sugars and amino acids and then convert them into acetic acid and other volatile fatty acids. *Longilinea* can metabolize a variety of carbohydrates, and *Anaerolinea* can metabolize a variety of carbohydrates to produce organic acids. The various hydrolytic - acidification strains were more abundant in A2 than in A3, and the abundances of *Ornatilinea* were 4.45% and 1.34%, respectively. The abundance of *Longilinea* was 2.22% and 0.67%, respectively. The reason for this analysis is that A2 primarily utilizes the organic matter in raw water as a carbon source. Although it has a hydrolysis acidification unit, owing to fluctuations in actual sewage quality, there may still be some complex organic matter that enters the system. This can lead to an increase in the abundance of some hydrolysis acidification functional bacteria. A3 primarily utilizes sodium acetate as a carbon source, so there were relatively few hydrolytic acidification functional bacteria present, while some common bacterial species were found at similar levels in these two reactors.

**Fig 10. The bacterial community structure of the denitrification unit before and after the AAOA system****.**

**Analysis of the community structure of nitrification units**

**Fig 11. The bacterial community structure of the nitrification unit in the AAOA system.**

As shown in Fig. 11, the nitrobacteria in O1, O2, and O3 primarily included *Nitrosomonas, Nitrobacter, Nitrospira,* and *Nitrospira*. The nitrobacteria in O2 were highly abundant. *Nitrosomonas* was the most abundant (25.36%), followed by *Nitrobacter* (7.44%), *Nitrospira* (2.67%) and *Nitrolancea* (2.62%). There was less *Nitrosomonas* in O1 (18.3%) than in O2. Some studies have found that high DO is more conducive to the enrichment of *Nitrosomonas* and *Nitrospira*. This finding is different from that in this study, and the reason may be that the small amount of organic matter in the A2 effluent may have an effect on the abundance of some nitrobacteria. The nitrobacteria in O3 were similarly abundant to those of O1 but less than that of O2. This may be owing to the high performance of O2, which leads to a low concentration of matrix that enters O3, The decrease in bacterial abundance under the action of long-term low load. As shown in Fig. XX, there were still aerobic denitrifying bacteria. *Paracoccus* was less abundant in O1, O2 and O3, although it was more abundant in O1 than in O2 and O3. Although the AAOA system has an A2 unit to utilize COD in raw water, a small amount of organic matter can enter the nitrification unit. Thus, there a small amount of aerobic denitrification will occur. As previously shown, the aerobic denitrification bacteria are present in immobilized inoculation sludge. The reason for the reduction in abundance is also related to the small role in the system. It was also shown that *Ignavibacterium* is less abundant in the system, while it is more abundant in O1 (0.35%) than in O2 and O3. These differences can also be attributed to the influent COD.

**Special thanks to you for all your valuable comments!**

Overall, we thank you for reconsidering our revised manuscript for publication in
**PLOS ONE**. We are looking forward to hearing from you soon.

We have attached the revised version of our manuscript for resubmission.

Once again, thank you very much for your comments and suggestions, and we hope that our manuscript now meets the high standards of you journal. Please contact us if there are any further questions.

Thank you and best regards.

Yours sincerely,
Jiawei Wang
E-mail: ykaisjz@163.com
